# Divergent Selection and Local Adaptation in Disjunct Populations of an Endangered Conifer, *Keteleeria davidiana* var. *formosana* (Pinaceae)

**DOI:** 10.1371/journal.pone.0070162

**Published:** 2013-07-22

**Authors:** Jing-Yu Fang, Jeng-Der Chung, Yu-Chung Chiang, Chung-Te Chang, Chia-Ying Chen, Shih-Ying Hwang

**Affiliations:** 1 Department of Life Science, National Taiwan Normal University, Taipei, Taiwan; 2 Division of Silviculture, Taiwan Forestry Research Institute, Taipei, Taiwan; 3 Department of Biological Sciences, National Sun Yat-Sen University, Kaohsiung, Taiwan; University of Massachusetts, United States of America

## Abstract

The present study investigated the genetic diversity, population structure, *F*_ST_ outliers, and extent and pattern of linkage disequilibrium in five populations of *Keteleeria davidiana* var. *formosana*, which is listed as a critically endangered species by the Council of Agriculture, Taiwan. Twelve amplified fragment length polymorphism primer pairs generated a total of 465 markers, of which 83.74% on average were polymorphic across populations, with a mean Nei’s genetic diversity of 0.233 and a low level of genetic differentiation (approximately 6%) based on the total dataset. Linkage disequilibrium and HICKORY analyses suggested recent population bottlenecks and inbreeding in *K. davidiana* var. *formosana*. Both STRUCTURE and BAPS observed extensive admixture of individual genotypes among populations based on the total dataset in various clustering scenarios, which probably resulted from incomplete lineage sorting of ancestral variation rather than a high rate of recent gene flow. Our results based on outlier analysis revealed generally high levels of genetic differentiation and suggest that divergent selection arising from environmental variation has been driven by differences in temperature, precipitation, and humidity. Identification of ecologically associated outliers among environmentally disparate populations further support divergent selection and potential local adaptation.

## Introduction

Natural environments are spatially and temporally heterogeneous. Divergent selection across environments may induce adaptive divergence, resulting in local adaptation [1], [2]. In consequence, populations in contrasting environments can evolve differences in phenotypic variation that are fitness-related [1]. Natural selection is expected to increase locally advantageous allele frequencies; therefore we can detect adaptive loci by identifying those that display higher genetic differentiation among populations when compared with the rest of the genome [3], [4]. Identification of adaptive genetic variation in populations of endangered species represents adaptive potential responding to natural selection, which is of particular concern to enable effective management of these rare species.

Geographically isolated populations of a species can serve as natural laboratories for studying adaptive processes [5]–[8]. Population genomic approaches are useful for identifying divergent selection among populations at specific loci, even in non-model organisms with little genetic information [9], [10]. In one approach, *F*_ST_-based population genomic methods can be employed to search for adaptive loci by scanning a large number of markers including dominant markers such as amplified fragment length polymorphisms (AFLPs) [11]. DFDIST and BAYESCAN are two commonly used approaches. For example, DFDIST [12] generates an expected neutral distribution of *F*_ST_ values under a classic symmetrical island model [13] and loci potentially under positive selection can be identified if they exhibit unusually high *F*_ST_ deviations from neutral estimates. However, DFDIST is prone to generating false positives when gene flow is asymmetrical across populations, and/or when a population has experienced a bottleneck. To address this problem, the BAYESCAN approach estimates population-specific *F*_ST_ values by considering different demographic histories and different amounts of genetic drift between populations [14]. It has also been suggested that BAYESCAN is more robust to false positives when using dominant markers [15].

An alternative approach to detecting adaptive variation is to correlate allelic frequencies with environmental gradients, in order to determine which ecological factors may have played a role in natural selection [16]–[18]. In this context, changes in allele frequencies can be interpreted as being driven by divergent adaptation due to differences in local environments [19]. This strategy has successfully been applied to identify candidate loci correlated with a variety of environmental conditions [7], [8], [16]–[23].

Taiwan cow-tail fir (*Keteleeria davidiana* (Franchet) Beissner var. *formosana*) is an endemic coniferous evergreen tree species. It is presently disjunctly distributed on rocky ridges of northern Taiwan at elevations of 300–600 m and of southern Taiwan at elevations of 500–900 m [24]. A progenitor-derivative relationship between *K. davidiana* (Bertrand) Beissner that occurs in China and *K. davidiana* var. *formosana* was proposed by Farjon [25]. Pollen of *Keteleeria* began to appear at the Plio-Pleistocene boundary and continued through the early Pleistocene Praetiglian in central Taiwan at an elevation of 550 m, but decreased to a minor proportion afterwards [26]. Pollen of the warmth-loving *Keteleeria* was also observed in very minor proportions or rarely during the late Pleistocene until the early phase of the Holocene around 10,000 years ago in central Taiwan [26]. The period during the late Pleistocene to Holocene exhibited expansion of grasses (Poaceae) and subtropical forests [27]. Present forests at elevations of 500–1500 m in Taiwan are dominated by subtropical species such as *Machilus* and *Castanopsis* [28]. It is likely that the recalcitrant seed storage behavior [29] and low natural regeneration rate [30] of *K. davidiana* var. *formosana* may have been a disadvantage in competing with rapidly growing subtropical forest species. Moreover, habitat destruction due to severe logging in the early 20th century caused substantial reductions in population sizes of this species in northern and southern Taiwan [31]. It is probable that populations of *K. davidiana* var. *formosana* in Taiwan were historically larger and more common in low-elevation forests than at present. Population bottlenecks might have occurred in this species and resulted in the loss of genetic variability, therefore we suspect that populations of this species may exhibit relatively high frequencies of linkage disequilibrium (LD). Moreover, present *K. davidiana* var. *formosana* populations occupy significantly different environmental niches and also vary in their floristic compositions [32]. Therefore, contemporary *K. davidiana* var. *formosana* populations in Taiwan may be ideal for the study of early characteristics of divergent selection evoked by environmental differences, leading to local adaptation [33]–[35].

In this study, we first determined the level of genetic diversity and partitioning of genetic variation among natural populations of *K. davidiana* var. *formosana* distributed in northern and southern Taiwan based on AFLPs. Contemporary population bottleneck and inbreeding were inferred based on LD and HICKORY [36] analyses. Estimation of the diversity measure, genetic structure, inbreeding, and the extent of LD can be used to determine whether *K. davidiana* var. *formosana* populations are genetically isolated, due to long-term demographic processes or recent bottlenecks inflicted by human disturbances. We also examined evidence for *F*_ST_ outliers in pairwise population comparisons using genome scan approaches (DFDIST and BAYESCAN). We then applied logistic regression methods including spatial analysis method (SAM) [17] and generalized estimating equations (GEE) [37] to test for associations between genetic data and environmental variables indicative of ecologically associated local adaptation.

## Materials and Methods

### DNA Materials and AFLP Genotyping

**Figure 1:**
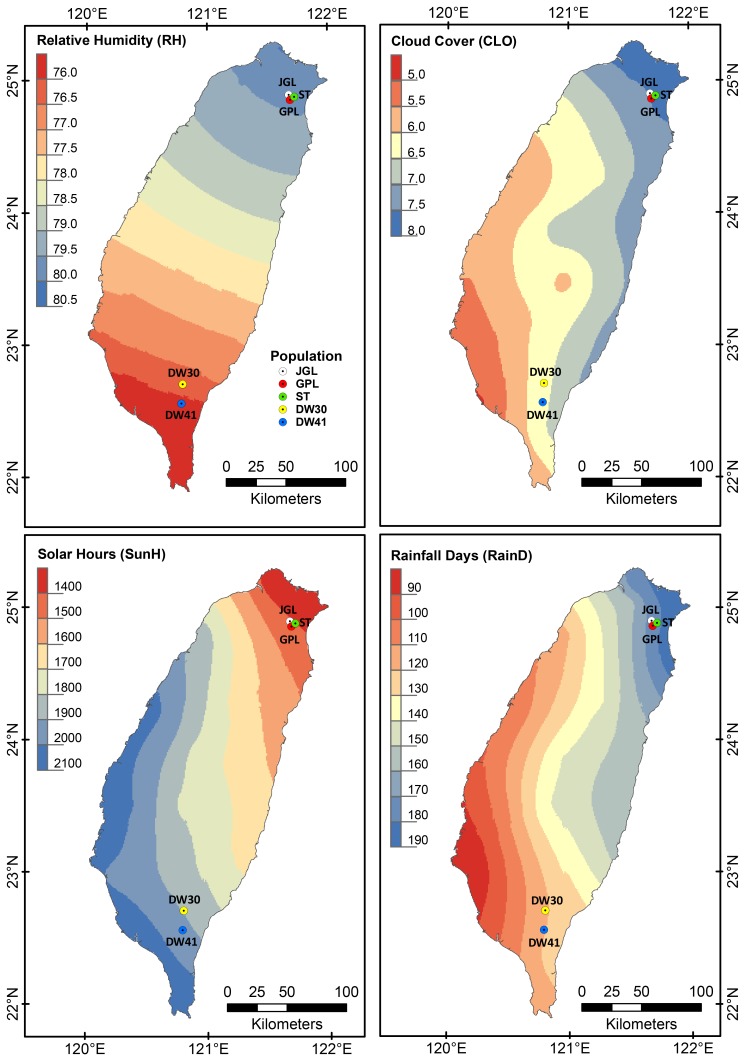
Sampling localities of *Keteleeria davidiana* var. *formosana* and annual mean gradients (color gradients) of four environmental variables. Significant differences among *K. davidiana* var. *formosana* populations were found in four environmental variables including relative humidity (RH), cloud cover (CLO), time of sunshine (SunH), and wet days (number of days with >0.1 mm rain per month; RainD) according to the Welch ANOVA (see Table S2). Annual mean gradients were smoothed using a universal spherical model of the Kriging method in ArcGIS (Carrera-Hernández and Gaskin 2007) [40].

We collected 163 individuals from northern Taiwan including sites at Jingualiao (JGL, *n* = 20), Gupoliao (GPL, *n* = 10), and Shihtsao (ST, *n* = 81), and from southern Taiwan including sites at Dawu 30 (DW30, *n* = 18) and Dawu 41 (DW41, *n* = 34) (Figure 1). All plant materials were collected with the approval of the Forestry Bureau, Council of Agriculture, Taiwan (permit number: 101-AgroScience-1.1.2-B-e1). Genetic analysis of AFLPs was performed for all collected samples following the general method of Vos *et al*. [38]. Total genomic DNA (200 ng) was digested with 5 U *Eco*RI and 5 U *Mse*I (Yeastern Biotech, Taipei, Taiwan). Digested DNA products were added to a 10-µl reaction mixture containing 0.5 µM of the *Eco*RI adaptor, 5 µM of the *Mse*I adaptor, and 30 U T4 DNA ligase (Yeastern Biotech) at 22°C for 1 h. Digested samples were diluted (1∶9 dilution with water), and 1 µl of each diluted sample was used as a template to perform pre-selective amplification in a 10-µl volume containing 1× PCR buffer (25 mM KCl, 10 mM Tris-HCL, 1.5 mM MgCl_2_, and 0.1% Triton X-100), 100 nM each of the *Eco*RI (E00: 5′-GACTGCGTACCAATTC-3′) and *Mse*I (M00: 5′-GATGAGTCCTGAGTAA-3′) primers, 0.25 mM dNTPs, and 1 U *Taq* DNA polymerase (Bernardo Scientific, Taipei, Taiwan). Conditions of the pre-selective amplification were initial holding at 72°C for 2 min and pre-denaturation at 94°C for 3 min, followed by 25 cycles of 30 s at 94°C, 30 s at 56°C, and 1 min at 72°C, with a final 5-min holding at 72°C. Selective amplification was performed using 12 *Eco*RI-*Mse*I selective primer combinations with sequences of the three bases additional to the E00 and M00 primers (Table S1), in which an *Eco*RI selective primer was labeled with fluorescent dye (6-FAM or HEX). Conditions of selective amplification were a 10-µl volume containing 1× PCR buffer, 100 nM of the *Eco*RI selective primer, 100 nM of the *Mse*I selective primer, 0.25 mM dNTPs, 0.75 U *Taq* DNA polymerase, and 1 µl diluted pre-selective amplified product (1∶9 dilution with water). The selective PCR amplification was performed with initial holding at 94°C for 3 min, followed by 13 cycles of 30 s at 94°C, 30 s at 65°C with 0.7°C touchdown per cycle, and 1 min at 72°C, then 23 cycles of 30 s at 94°C, 30 s at 56°C, and 1 min at 72°C, with a final 5-min holding at 72°C.

Amplified fragments were separated by electrophoresis on an ABI PRISM 3100 sequencer (Applied Biosystems, Foster City, CA, USA). Electropherograms were automatically analyzed for fragment presence/absence in each individual in the range of 50–500 bp with a GeneMapper v3.7 setting of a fluorescent signal detection threshold of 100 units to avoid background noise. All loci of all individuals were rechecked to remove loci with low peaks and loci separated by less than one nucleotide. The genotyping error rate per locus was calculated as the ratio of mismatches in 30 replicates to the total number of replicated markers. The error rate for 465 markers (21–61 per primer combination) was estimated to be 3.8% (Table S1), which was lower than the maximum error of 10% suggested by Bonin *et al.* [39]. AFLP genotype data were deposited at Dryad: http://dx.doi.org/10.5061/dryad.7 cm6b.

### Environmental Data

We obtained data for 11 environmental variables recorded from 1990–2011 at 390 meteorological stations from the Central Weather Bureau, Taiwan. Environmental variables included mean temperature (Tmean), maximum temperature (Tmax), minimum temperature (Tmin), mean wind speed (WSmean), precipitation (PRE), relative humidity (RH), cloud cover (CLO), time of sunshine (SunH), days of maximum temperature >30°C (D_30_), days of minimum temperature <10°C (D_10_), and wet days (number of days with >0.1 mm of rain per month, RainD). Values of the 11 environmental variables at localities of *K. davidiana* var. *formosana* investigated were obtained by interpolation of nearby meteorological observations, resulting in a 1 km^2^ grid resolution using a universal spherical model and the Kriging method in ArcGIS [40]. Differences in the 11 environmental variables among all populations investigated were tested using a multivariate analysis of variance (MANOVA) based on the Pillai-Bartlett statistic. Differences in each environmental variable among populations were further analyzed using the Welch ANOVA implemented in JMP v7.0 [41].

### Genetic Diversity and Structure

AFLP profiles of band presence (1) and absence (0) were scored and used to calculate unbiased Nei’s genetic diversity (U*H*_E_) [42] using AFLP-SURV v1.0 [43] under the assumption of Hardy-Weinberg equilibrium. Only loci with frequencies ranging 0.05–0.95 were considered polymorphic for U*H*_E_ calculations.

Total AFLP, outlier, and neutral locus datasets were used to estimate genetic differentiation via several approaches. First, pairwise genetic differentiation (*F*_ST_) between populations was calculated using ARLEQUIN v3.5 [44]. Second, total variation was partitioned into within-population, among-population, among population within-regions, and between-region components using analysis of molecular variance (AMOVA) with 50,000 permutations implemented in ARLEQUIN. Population structure was inferred using Bayesian clustering methods implemented in STRUCTURE v2.3 [45], [46] and BAPS v5.3 [47], [48]. STRUCTURE considers allele frequencies and LD information to infer the most likely individual clusters and admixtures of genotypes. BAPS infers the most likely individual clusters considering populations as sampling units, relying on differences in allele frequencies to partition individuals rather than LD [49], and is thought to be more powerful in identifying hidden structures within populations [50]. In STRUCTURE, we assumed an admixture model with an informative prior of sampling location. Ten replicates were performed for each level of *K* ( = 1–5) with 10^6^ iterations and 10^5^ burn-in steps. The mean log probability, (*LnP*(*D*)) [45], and the change in the log probability, Δ*L*(*K*) [51], were used to evaluate the fit of different clustering scenarios. In BAPS, the maximum number of groups (*K*) was set to 2–4 according to results obtained from the mean log probability analyzed by STRUCTURE. Each run was replicated three times, and the results were averaged according to the resultant likelihood scores.

The Bayesian program, HICKORY v1.1 [36], was used to examine whether non-random mating occurred in *K. davidiana* var. *formosana* populations. HICKORY directly estimates an *F*_ST_ analogue (*θ* ^B^) from dominant markers, and is unaffected by Hardy-Weinberg and inbreeding coefficient (*f*) assumptions [52]. Four HICKORY models were fitted to the total AFLP dataset including (i) the full model that allows for inbreeding and calculates both *f* and *θ* ^B^; (ii) the *f* = 0 model that assumes no inbreeding and calculates *θ* ^B^; (iii) the *θ* ^B = ^0 model that implies no differentiation between populations and calculates *f*; and (iv) the *f*-free model that chooses *f* at random from a prior distribution and calculates *θ* ^B^ separately during a Monte Carlo Markov chain (MCMC) run. In the full and *θ* ^B = ^0 models, a uniform prior distribution of *f* was assumed. Separate analyses of the population structure, including within-population, among-population within regions, and between-region components, were performed in HICKORY. The Deviance Information Criterion (DIC) was used to choose a best fitting model. Caution must be taken if the difference in DIC between models is smaller than six units [52]. A lower Dbar+pD (model fit+model complexity) can be used to ensure that one model is favored over another based not merely on a difference in model complexity [53]. HICKORY settings (burn-in = 50,000, samples = 250,000, thinning = 50) were used for sampling and chain length parameters. The analyses were run twice in order to check the convergence of parameters.

### Identification of Outliers and Tests for Associations between Genetic Data and Environmental Variables

We performed pairwise population comparisons for signatures of selection using the genome scan methods DFDIST and BAYESCAN. In DFDIST, allele frequencies are estimated from the proportion of recessive phenotypes in the sample based on the Bayesian approach by Zhivotovsky [54]. A neutral distribution of *F*_ST_ conditioned on heterozygosity was generated using 10^5^ iterations of coalescent simulations in a symmetrical island migration model at migration-drift equilibrium. To estimate the empirical multilocus *F*_ST_, the highest and lowest 30% of the initial *F*_ST_ calculated for all AFLP loci were removed when calculating the mean *F*_ST_. A locus with an unusually high *F*_ST_ value conditioned on heterozygosity was considered an outlier potentially under positive selection. Significant *P* values of outlier loci were evaluated at the 95% confidence level by applying a false discovery rate (FDR) of 5% [55]. The Bayesian likelihood method in BAYESCAN, which compares a model of selection versus neutrality via a reversible-jump MCMC, was also used to detect outliers [14]. In BAYESCAN, a Bayes factor (BF) representing the posterior probability ratio of selection over neutrality is calculated. Following 100 pilot runs of 50,000 iterations, a sample size of 50,000 and thinning interval of 20 among the 10^6^ iterations (with 10^5^ burn-in) were used to identify loci potentially under selection. In this study, a locus with log_10_(BF) >0.5 was considered evidence for selection applying Jeffreys’ scale of evidence [56].

To search for ecologically associated outliers, direct associations between allelic frequencies of all AFLP loci and values of environmental variables were tested using a univariate logistic regression method implemented in SAM [17], [57]. All possible pairwise combinations of alleles vs. environmental variables were considered. *P* values of Wald tests with Bonferroni adjustment (α = 0.05) for multiple comparisons were used to evaluate the significance of the associations. However, Joost *et al*. [17] cautioned on use of correlative approach in SAM because most environmental variables are correlated to some extent. A principal component analysis (PCA) was therefore used to examine correlations between environmental variables. The resultant PCs, which explained most of the variance, were used to test for associations with the genetic data by GEE method implemented in the R package GEEPACK [37]. GEE considers autocorrelation between individuals (response variables) collected at the same sampling location that would violate the independent assumption of response variables, and add a variance component to deal with correlated data. Logistic regression with a logit-link and binomial error distribution in GEE was applied to test for associations between AFLP loci and PCs. *P* values of Wald tests with Bonferroni correction (α = 0.05) were used to compare the null model (the PC effect restricted to 0) with the model which included one predictor variable (PC) to evaluate the significance of adding that individual predictor to the null model.

### Linkage Disequilibrium Analyses

Multilocus v1.3 [58] was used to perform LD analysis in order to draw inferences regarding lack of shared polymorphisms in *K. davidiana* var. *formosana* populations, indicative of a possibility of inbreeding. We calculated the association of alleles at different loci within each population for indices of association *I*_A_ [59]–[61], and its modified measure, *r*_d_, independent of number of loci [58]. Significance of observed *I*_A_ and *r*_d_ values was tested against 1000 permuted datasets, randomly reshuffled alleles across individuals among populations that were independently compared for each locus, in which sexual recombination was imposed. We further tested for LD based on the distribution of allelic mismatches between pairs of genotypes over all loci using an exact test implemented in ARLEQUIN.

## Results

### Population Genetic Diversity

Twelve primers generated a total of 465 AFLP loci in the entire sample with an overall repeatability of 96.2% (Table S1). The proportion of polymorphic loci ranged from 71.8% (DW41) to 98.5% (DW30) with an average value of 83.74% (Table 1). The level of U*H*_E_ averaged 0.233 and ranged from 0.197 (ST) to 0.312 (DW30) (Table 1). The mean U*H*_E_ was higher for southern populations (mean U*H*_E_ = 0.259) than northern populations (mean U*H*_E_ = 0.216), but the difference was not significant (Wilcoxon two-sample test, *P* = 0.80). Populations JGL, GPL, ST, and DW41 had U*H*_E_ values of 32.7%, 22.9%, 37.0%, and 34.2% lower than the U*H*_E_ of population DW30.

**Table 1:** Sampled populations, number of individuals, locality, level of polymorphism, genetic diversity, and linkage disequilibrium (LD) detected with the AFLP markers in *Keteleeria davidiana* var. *formosana*.

Population	Number of individuals	Latitudelongitude	Percentage ofpolymorphic loci (%)	Nei’s gene diversity(U*H*_E_) (SE)	*I*_A_	*r*_d_	Proportion of significant LD between AFLP loci (%)
Jingualiao (JGL)	20	24°54′52.278′′N121°40′36.679′′E	80.6	0.2103 (0.0055)	16.5918*	0.0390*	5.79
Gupoliao (GPL)	10	24°53′52.959′′N121°41′13.058′′E	85.2	0.2409 (0.0062)	20.0117*	0.0521*	9.75
Shihtsao (ST)	81	24°53′35.246′′N121°41′48.236′′E	82.6	0.1970 (0.0051)	25.0027*	0.0555*	6.12
Dawu 30 (DW30)	18	22°36′42.394′′N121°0′19.435′′E	98.5	0.3127 (0.0053)	6.5746*	0.0147*	2.75
Dawu 41 (DW41)	34	22°25′38.369′′N120°51′3.006′′E	71.8	0.2057 (0.0070)	10.9647*	0.0276*	6.09

*I*_A_, index of association; *r*_d_, modified index of association.

* *P*<0.001.

The proportion of significant LD between AFLP loci was evaluated applying a false discovery rate of 5%.

### Environmental Heterogeneity

The MANOVA showed significant environmental differences in habitats of *K. davidiana* var. *formosana* based on the Pillai-Bartlett statistic (V = 1.7859, F_44, 192_ = 3.5199, *P*<1.03e-09). Moreover, significant differences in the four environmental variables of RH, CLO, SunH, and RainD among populations were found using the Welch ANOVA (Figure 1, Table S2).

### Population Differentiation

Because similar levels of genetic differentiation, as analyzed either by pairwise *F*_ST_ or AMOVA, were found for the total and neutral datasets, we only report results based on the total and outlier datasets (47 outliers, see the following “outlier detection” section). Overall respective pairwise *F*_ST_ values were 0.0607 and 0.1997 for the total and outlier datasets. Pairwise *F*_ST_ was significantly greater than zero when comparing populations of the north to the south (average pairwise *F*_ST_ = 0.0778 and 0.2649, respectively for the total and outlier datasets) and also significantly greater than zero when comparing the two southern populations (pairwise *F*_ST_ = 0.0941 and 0.2689, respectively for the total and outlier datasets) after applying the Bonferroni correction at *P*<0.05 (Table 2). Pairwise *F*_ST_ values were low and not significantly greater than zero when the three northern populations were compared (average pairwise *F*_ST_ = 0.0153 and 0.0462, respectively for the total and outlier datasets). The majority of genetic variation was partitioned within-population for one- or two-regional groups in the AMOVA based on the total (93.68% or 92.49%, respectively) and outlier datasets (79.31% or 75.19%, respectively) (Table 3). The AMOVA also showed a low and non-significant level of genetic differentiation among the three northern populations using the total dataset (*Φ*_ST_ = 0.01088, *P* = 0.0549), but was significant using the outlier dataset (*Φ*_ST_ = 0.03672, *P* = 0.00138). However, the AMOVA showed significant genetic differentiation between the two southern populations for both the total and outlier datasets (*Φ*_ST_ = 0.09419 and *Φ*_ST_ = 0.26899, respectively, *P*<0.001). Genetic variation partitioned among populations was also significant respectively for the total and outlier datasets in both the one- (*Φ*_ST_ = 0.06316 and *Φ*_ST_ = 0.20694, *P*<0.001) and two-regional groups (*Φ*_ST_ = 0.07513 and *Φ*_ST_ = 0.24807, *P*<0.001). However, the level of genetic differentiation between the northern and southern regional groups was not significant according to the AMOVA result (*Φ*_CT_ = 0.03554, *P* = 0.0988 and *Φ*_CT_ = 0.14422, *P* = 0.0987, respectively for the total and outlier datasets).

**Table 2:** Pairwise *F*_ST_ estimates among five populations of *Keteleeria davidiana* var. *formosana.*

Population	JGL	GPL	ST	DW30
JGL				
GPL	0.02144 (0.06679)			
ST	0.00513 (0.03066)	0.01952 (0.04116)		
DW30	0.07659* (0.24700*)	0.04303* (0.19411*)	0.09267* (0.26299*)	
DW41	0.07454* (0.31518*)	0.09943* (0.30788*)	0.08083* (0.26274*)	0.09419* (0.26899*)

Values before and within the parentheses respectively represent estimates from total and outlier data.

See Table 1 for population abbreviation codes.

* *P*<0.0001 after Bonferroni correction at α = 0.05.

**Table 3:** Summary of the analysis of molecular variance based on total and outlier data of 163 samples of *Keteleeria davidiana* var. *formosana* individuals.

Source of variation	df	Percent of variation	*Φ*-Statistics	*P*-value
One group (all five populations)				
Among populations	4	6.32 (20.69)		
Within populations	158	93.68 (79.31)	*Φ*_ST_ = 0.06316 (0.20694)	<0.001 (0.001)
Total	162			
Two groups (north vs. south)				
Among groups	1	3.55 (14.42)	*Φ*_CT_ = 0.03554 (0.14422)	= 0.0988 (0.0987)
Among populationswithin groups	3	3.96 (10.39)	*Φ*_SC_ = 0.04105 (0.12136)	<0.001 (0.001)
Within populations	158	92.49 (75.19)	*Φ*_ST_ = 0.07513 (0.24807)	<0.001 (0.001)
Total	162			
North region				
Among populations	2	1.09 (3.67)		
Within populations	108	98.91 (96.33)	*Φ*_ST_ = 0.01088 (0.03672)	= 0.0549 (0.00138)
Total	110			
South region				
Among populations	1	9.42 (26.90)		
Within populations	50	90.58 (73.10)	*Φ*_ST_ = 0.09419 (0.26899)	<0.001 (0.001)
Total	51			

Values before and within the parentheses respectively represent estimates from total and outlier data.

Using the total dataset, Bayesian HICKORY analyses were used to evaluate the contemporary reproductive mode of *K. davidiana* var. *formosana*. The full model had the best fit to the data based on DIC value (Table 4) [36]. Although the difference in DIC values was only 1.31 units between the full and *f = *0 model for the northern population group, the smaller Dbar+pD of the full rather than the Dbar+pD of the *f* = 0 model ensured that the full model was also the best fitting model for this population group [53]. Estimates of *f* were extremely large for both the full and *θ* ^B^ = 0 models (>0.96 for all hierarchical population groups). It is known that estimates of *f* derived from dominant marker data can be unrealistically large [36]. In HICKORY, the *f*-free model was implemented to deal with unreliable *f* estimates by selecting *f* values from a uniform prior distribution without generating a posterior distribution of *f*, and gave an estimate of *f* around 0.5 for all hierarchical population groups (Table 4). This result suggests that a certain degree of inbreeding was likely to have played an important role in shaping the contemporary gene pool structure of *K. davidiana* var. *formosana*. Moreover, under the full model, *θ* ^B^ = 0.0567 (95% confidence interval (CI): 0.051–0.063) was determined to be an unbiased Bayesian estimate of genetic differentiation among all populations. Between regions, the Bayesian *θ* ^B^ estimate was 0.0454 (95% CI: 0.038–0.053). Bayesian *θ* ^B^ estimates were 0.0045 (95% CI: 0.001–0.009) and 0.0997 (95% CI: 0.083–0.118) for genetic differentiation among populations respectively within the northern and southern regions, under the full model. The *θ* ^B = ^0 model had a substantially larger DIC than that of the full model, indicating good evidence for genetic differentiation among populations in all hierarchical population groups. However, only a very small amount of genetic variation was partitioned among populations within the northern region, similar to the pairwise *F*_ST_ and AMOVA results.

**Table 4:** Genetic structure analysis of the total data using HICKORY v1.1 (Holsinger and Lewis 2003) [36].

Modelparameter	Within populations	Between regions	Within regions	
			North	South
Full model				
* θ* ^B^	0.0567 (0.051–0.063)	0.0454 (0.038–0.053)	0.0045 (0.001–0.009)	0.0997 (0.083–0.118)
Dbar	9281.59	4817.73	5666.89	3666.23
Dhat	7905.07	4046.34	5160.75	2997.47
pD	1376.53	771.387	506.146	668.762
DIC	10658.1	5589.12	6173.04	4334.99
f	0.9862 (0.949–1.000)	0.9868 (0.953–1.000)	0.9681 (0.873–0.999)	0.9619 (0.859–0.999)
*f* = 0 model				
* θ* ^B^	0.0323 (0.029–0.036)	0.0256 (0.021–0.030)	0.0022 (0.001–0.004)	0.0589 (0.048–0.071)
Dbar	9297.70	4832.88	5673.53	3670.41
Dhat	7912.63	4056.59	5172.70	2993.83
pD	1385.07	776.29	500.82	676.57
DIC	10682.8	5609.17	6174.35	4346.98
*θ* ^B = ^0 model				
Dbar	12174.9	6384.54	5760.98	4802.75
Dhat	11724.4	5933.71	5317.77	4385.74
pD	450.523	450.838	443.211	417.015
DIC	12625.4	6835.38	6204.19	5219.77
f	0.9958 (0.985–1.000)	0.9957 (0.984–1.000)	0.9960 (0.985–1.000)	0.9947 (0.981–1.000)
*f*-free model				
* θ* ^B^	0.0642 (0.047–0.090)	0.0725 (0.045–0.116)	0.0303 (0.018–0.051)	0.1123 (0.084–0.145)
Dbar	9597.77	5294.96	5713.24	3795.45
Dhat	7725.97	4052.43	4685.70	2981.38
pD	1871.80	1242.53	1027.54	814.066
DIC	11469.6	6537.49	6740.78	4609.51
f	0.4941 (0.023–0.976)	0.506 (0.028–0.974)	0.4974 (0.023–0.976)	0.5008 (0.024–0.979)

Values for sampling and chain length parameters for computations were as follows: burn-in 50,000; sample 250,000; thinning 50. See text for further explanations of the four models and the model selection criteria.

*θ* ^B^, is the best Bayesian inference estimate of the proportion of genetic diversity due to differences among contemporaneous populations, and is an analogue to *F*_ST_.

Dbar, is −2 times the mean posterior log likelihood and is a measure of how well the model fits the data (smaller values indicate a better fit).

Dhat, is −2 times the log likelihood evaluated at the posterior mean, and it gives a measure of how well the best point estimate fits the data.

pD, is a measure of model complexity, i.e., the effective number of parameters being estimated (pD = Dbar - Dhat).

DIC, deviance information criterion.

f, an estimate of *F*_is_, inbreeding within a population.

**Figure 2:**
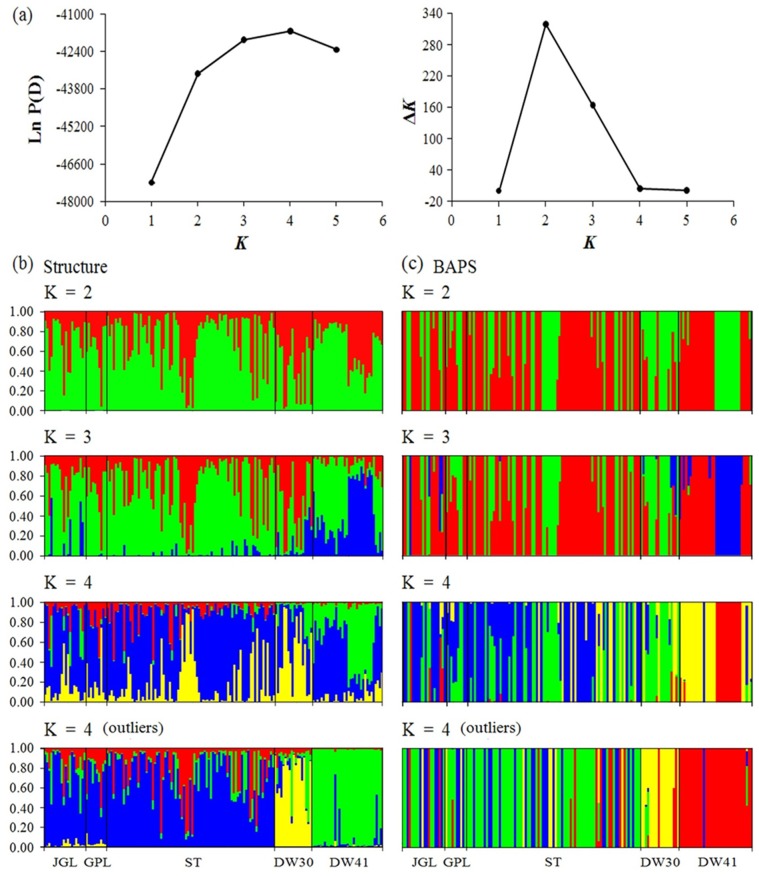
STRUCTURE and BAPS inferences representing assignments of individual genotypes to five *Keteleeria davidiana* var. *formosana* populations based on AFLPs. (a) Log likelihood, *LnP(D)*, and changes in the log likelihood, Δ*L*(*K*), for different scenarios of groupings respectively using STRUCTURE (Pritchard 2000) [45] and Evanno *et al*. (2005) [51]. (b) Bar plots represent STRUCTURE inferences of individual assignments (*K* = 2–4). (c) The bar plots represent BAPS inferences of individual assignments (*K* = 2–4). Each color represents the most likely ancestry of the cluster from which the genotype or partial genotype was derived. Each vertical bar represents one individual multilocus genotype. In total, 465 AFLP loci were used in the analysis except in the bottom panel of the illustration labeled “outliers”, which indicates analyses based on 47 outliers only. Multiple colors within a vertical bar indicate admixtures of genotypes from different clusters. Populations are separated by black lines.

In the STUCTURE analysis based on the total dataset, the maximal Δ*K* value occurred at *K* = 2 (Figure 2a). However, the highest mean log likelihood was obtained when *K* = 4 (Figure 2a). With differentiation of the two clusters according to Δ*K* = 2, neither STRUCTURE nor BAPS provided a prominent phylogeographic break among populations (Figure 2b, c). Bar plots showed varying extents of admixture among populations in various clustering scenarios (Figure 2b, c). The BAPS result at *K* = 4, based on the total dataset, indicated a distinction of the southern DW41 population from all other populations, and also genetic substructuring within this population (Figure 2c). When the outlier dataset was used in the STRUCTURE and BAPS analyses at *K* = 4, clear distinctions of disjunctly distributed northern and southern populations and between the two southern populations were observed. However, extensive admixtures of individual genotypes among the three northern populations, between regions and between the two southern populations were also observed, but no genetic substructuring was found within the southern DW41 population (Figure 2b, c).

### Outlier Detection

We performed pairwise population comparisons to identify *F*_ST_ outliers using the DFDIST and BAYESCAN methods. To minimize the presence of false positives, we considered an AFLP locus to have greater support for being an outlier if it was identified by both the DFDIST and BAYESCAN methods. In total, 47 AFLP loci were detected as outliers by both the DFDIST and BAYESCAN methods for ten pairwise population comparisons. Because different sets of loci were detected as potentially being under selection in separate pairwise population comparisons, we present only results that are important in light of inferring adaptive divergence and local adaptation.

**Figure 3:**
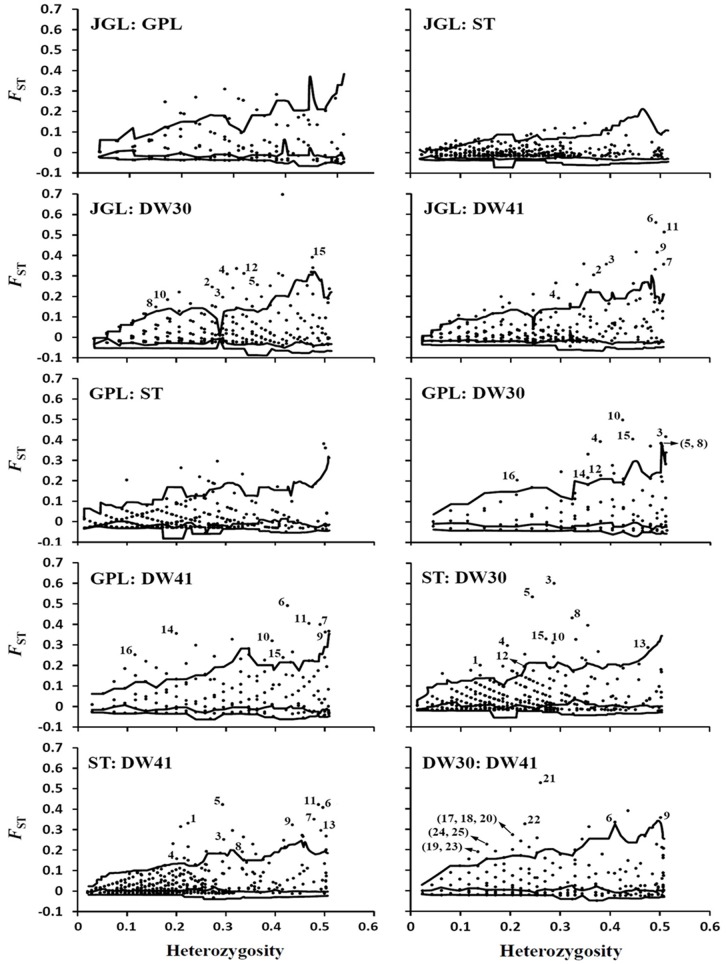
Genome scan to identify selective outlier loci with the DFDIST approach. Plots represent pairwise comparisons of the five *Keteleeria davidiana* var. *formosana* populations investigated. The lower, intermediate, and higher lines respectively represent the 2.5%, 50%, and 97.5% confidence intervals. AFLP loci above the 97.5% line are regarded as outlier loci under positive selection.

**Figure 4:**
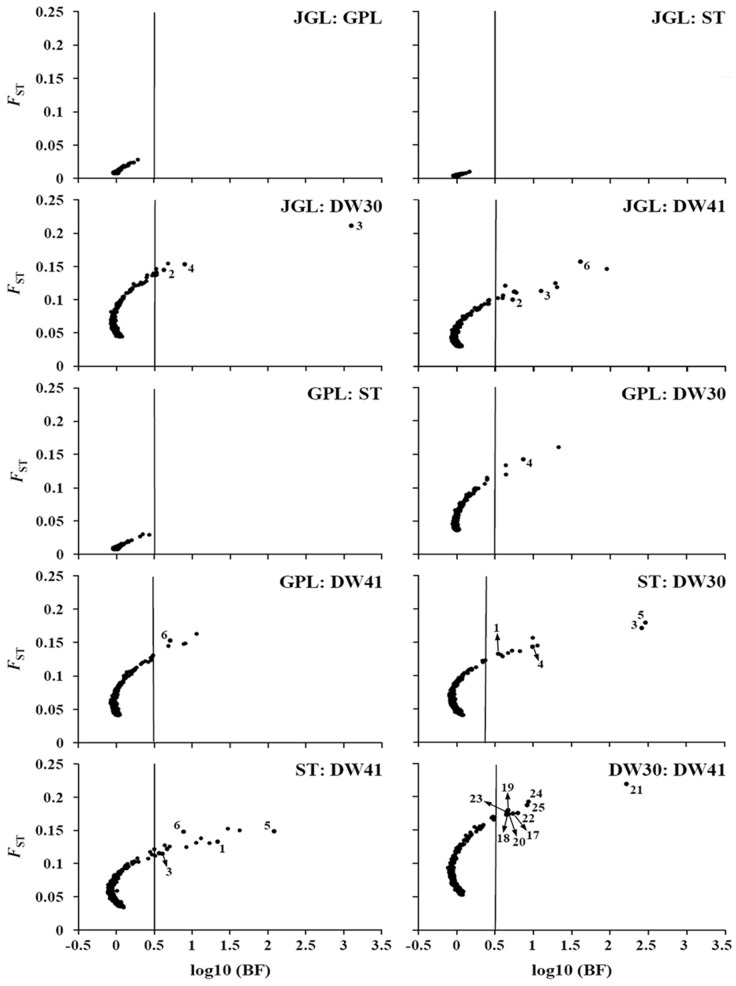
Genome scan to identify selective outlier loci with the BAYESCAN approach. Plots represent pairwise comparisons for the five *Keteleeria davidiana* var. *formosana* populations investigated. *F*_ST_ represents locus-specific genetic divergence between populations; log_10_BF (log_10_ Bayes factor) represents a decision factor on a logarithmic scale (base 10) to determine selection; and the vertical line indicates “substantial” evidence for selection according to the scale of evidence suggested by Jeffreys (1961) [56].

When the three northern populations were compared, many outliers were identified using DFDIST while none were detected using BAYESCAN (Figures 3, 4, Table S3). Therefore, no outliers were inferred with high confidence when the three northern populations were compared.

Using DFDIST, outliers were commonly identified in all comparisons between the southern DW30 population and the three northern populations, and between the southern DW41 population and the three northern populations (Figure 3, Table S3). Outliers were also commonly identified when either southern populations was compared independently to the northern JGL, GPL, and ST population, respectively (Figure 3, Table S3). Using BAYESCAN, only one outlier (locus 4) was commonly detected in all comparisons between the southern DW30 population and the three northern populations (Figure 4, Table S3). Similarly, one locus (locus 6) was commonly detected as an outlier in all comparisons between the southern DW41 population and the three northern populations. There were two (loci 2 and 3), zero, and three (loci 1, 3, and 5) loci that were respectively detected as being common outliers when either southern population was compared to the northern JGL, to the northern GPL, and to the northern ST population.

AFLP loci 1–6 were repeatedly detected as being outliers by both the DFDIST and BAYESCAN approaches in various southern vs. northern population pair comparisons. In addition, nine AFLP loci (loci 17–25) were detected as outliers by both DFDIST and BAYESCAN when comparisons were made between the two southern populations (Figures 3, 4, Table S3). Among the outliers identified in multiple comparisons of geographically distant populations by both DFDIST and BAYESCAN, SAM found four of them (loci 1, 3, 4, and 5) to be strongly associated with environmental variables SunH, RH, CLO, and RainD (adjusted *P*-value <10^−7^, Table S3).

PCA analysis showed that environmental variables are highly correlated with the first two axes explained 99.54% (89.70% and 9.84%, respectively for PC1 and PC2) of the total variation. Increasing the value of PC1 increased values of Tmean, Tmax, Tmin, SunH, and D_30_, but decreased values of PRE, RH, CLO, RainD, and D_10_. Increasing the value of PC2 increased value of WSmean. GEE method found independent sets of 54 and 34 AFLP loci significantly associated with the PC1 and PC2, respectively (adjusted *P*-value <10^−6^) (Table S3). Three loci (loci 1, 4, and 5) strongly associated with PC1 were also detected as being outliers by all other methods including DFDIST, BAYESCAN, and SAM. Therefore, loci 1, 4, and 5 can be considered potential candidates of ecologically associated outliers correlated with temperature, precipitation, and humidity.

### Linkage Disequilibrium

Multilocus LD was assessed using the *I*_A_ and *r*_d_ indices, and it was found to be statistically greater than expected under a null distribution in all populations (Table 1), indicating inbreeding within *K. davidiana* var. *formosana* populations. In a total of 107,880 locus-pair comparisons, using the two-locus gametic disequilibrium exact test, 11.54% were detected to have significant LD between AFLP loci (applying an FDR of 5%) at the total population level. The proportion of significant pairwise LD averaged 6.10% and varied from 2.75% (DW30) to 9.75% (GPL). Moreover, patterns of LD between ecologically and non-ecologically associated outliers substantially varied among *K. davidiana* var. *formosana* populations (Table 5).

**Table 5:** Two-locus linkage disequilibrium test for associations between ecologically and non-ecologically associated outliers within each of the five *Keteleeria davidiana* var. *formosana* populations.

AFLP lcous	Population				
	JGL	GPL	ST	DW30	DW41
1		14*	3		101
2			5		
3	5*				4
4	8*		8, 17	21*	
5	4*	101*	72		
8					4
15		5*			
17			1		
19			1		
20			1	1	
21			1,4		
23			5		
78		5*			
95		5*			
189	4*				
208			1*		4*
269		5*			
279					4*
388	4*	1*			

Population abbreviation codes are explained in Table 1.

AFLP loci 1, 4, and 5 are ecologically associated outliers according to the results analyzed by the spatial analysis method (SAM) (Joost *et al*. 2007, 2008) [17], [57] and generalized estimating equations (Halekoh *et al*. 2006) [37]. The rest of AFLP loci are outliers that are not ecologically associated.

All loci were significant after the false discovery rate correction at *P*<0.05.

* Significant after Bonferroni correction at *P*<0.002.

## Discussion

The total AFLP data revealed a relatively high level of genetic diversity at the species level. The low level of genetic differentiation suggests that historically, *K. davidiana* var. *formosana* had a larger population size and/or a more-widespread distribution than at present. Population size reductions due to recent anthropogenic disturbances may have generated significant within-population LD, and exhibited greater impacts on levels of genetic diversity in the JGL, GPL, ST, and DW41 populations than in the DW30 population. Extensive admixture within individual genotypes found between populations of the northern and southern regions based on the total dataset, contrary to what would be expected of geographically isolated populations assumed to have become highly differentiated [62], may have resulted from incomplete lineage sorting. However, high levels of genetic differentiation were found based on the outlier dataset. LD and HICKORY results indicate a potential recent genetic bottleneck and inbreeding. Divergent selection between the geographically isolated northern and southern populations and also between the geographically neighboring southern DW30 and DW41 populations at outlier loci is likely due to strong selective forces acting even in the face of significant levels of gene flow. Our results of the same ecologically associated outliers in the two southern populations indicate that temperature, precipitation, and humidity may have been contributing factors in the evolution of *K. davidiana* var. *formosana*. However, local adaptation can be dampened by the homogenizing effect of gene flow, which prevents population differentiation [63], [64] unless strong selection maintains differentially adapted alleles.

### Genetic Diversity and Linkage Disequilibrium

The average U*H*_E_ value was higher in the warm-associated *K. davidiana* var. *formosana* (average U*H*_E_ = 0.233) than the average U*H*_E_ ( = 0.184) value of a temperate coniferous species *Cunninghamia konishii* also endemic to Taiwan based on an AFLP analysis [65]. The average U*H*_E_ was also higher in *K. davidiana* var. *formosana* compared to the average U*H*_E_ of other Pinaceae species such as *Cedrus atlantica* (average U*H*_E_ = 0.129) endemic to Morocco and Algeria [66] and the endangered *Abies ziyuanensis* (average U*H*_E_ = 0.136) that occurs in China [67]. However, the average *H*_E_ of *K. davidiana* var. *formosana* was lower than the average U*H*_E_ ( = 0.283) of a widespread Pinaceae species *Pinus contorta* that occurs in North America [68]. Nevertheless, the level of genetic diversity of *K. davidiana* var. *formosana* was close to the average value for 13 plant species in general (average *H*_E_ = 0.230) based on AFLPs, and also close to the average values for 37 long-lived perennials (average *H*_E_ = 0.250) and seven wind and/or water-dispersed species (average *H*_E_ = 0.270) based on random amplified polymorphic DNAs [69]. Genetic variation is an important resource for populations and/or species to adapt to changing environmental conditions [70], [71]. Populations of *K. davidiana* var. *formosana*, although distributed in restricted areas of northern and southern Taiwan, appear to still harbor a large proportion of variation from ancestral populations and can likely provide substantial amounts of genetic variation for evolutionary dynamics under natural selection [72], [73].

Significant LD was detected in all populations according to *I*_A_, *r*_d_, and the proportion of significant LD between AFLP loci, although LD is known to decay rapidly over time at the genome-wide and within-gene levels in Pinaceae [74]–[77]. In some cases, extensive intergenic LD was also found in Pinaceae species [76], [77]. Based on predictions from population genetic theory, large populations under mutation-drift equilibrium tend to have low levels of LD, whereas genetic drift can cause high LD in small populations [78]. Retention of significant LD indicates recent population bottlenecks in *K. davidiana* var. *formosana* that probably have not had sufficient time to significantly decay. However, other factors such as the ages of the mutations, natural selection, and recombination rates can also influence the degree of LD [79]–[81]. Nonetheless, our results demonstrated significant LD between AFLP loci having a genome-wide effect, suggesting that it is a consequence of recent population bottlenecks [82], [83]. Although population size declines might not have significantly affected the amount of genetic variation of the southern DW30 population, the result of significant LD conformed to the expectation of a relatively stronger impact on LD than on the level of diversity due to a bottleneck [84]. However, population size reductions may have greater impacts on all other populations because of their lower levels of U*H*_E_ compared to the DW30 population.

### Population Structure, Incomplete Ancestral Lineage Sorting, and Genetic Isolation

In the pairwise *F*_ST_ and AMOVA results, similar patterns of genetic differentiation analyzed in all hierarchical population groups using both the total and outlier datasets were found. In light of comparisons of our findings with other studies, we focus discussion on results obtained from the total dataset if not indicated otherwise. The level of genetic differentiation of *K. davidiana* var. *formosana* was much lower based on AFLPs, according to pairwise *F*_ST,_ AMOVA, and HICKORY results, than the level of genetic differentiation of another endemic coniferous species *Cun. konishii* (*Φ*_ST_ = 0.246) that occurs in Taiwan [65]. The level of genetic differentiation in *K. davidiana* var. *formosana* was also much lower than that in other Pinaceae species such as the disjunctly distributed *Ced. atlantica* (*F*_ST_ = 0.25) [66] and *A. ziyuanensis* (*F*_ST_ = 0.482, *Φ*_ST_ = 0.55) [67]; but was higher than the level of genetic differentiation in the widespread *P. contorta* (*F*_ST_ <0.02, *Φ*_ST_ = 0.016) [68]. Nevertheless, the level of genetic differentiation in *K. davidiana* var. *formosana* based on AFLPs was in accordance with low levels of genetic differentiation generally found for coniferous species using allozymes and microsatellites [85], [86].

In a simulation study by Latch *et al*. [87], both STRUCTURE and BAPS performed well in inferring the number of clusters at *F*_ST_ around 0.03, which was smaller than the overall pairwise *F*_ST_ of *K. davidiana* var. *formosana*. However, both STRUCTURE and BAPS observed extensive admixture of individual genotypes across populations, especially at *K* = 2 and 3, within regions and between the two geographically isolated regions of *K. davidiana* var. *formosana*. The admixture of individual genotypes among populations might have resulted from incomplete lineage sorting of ancestral variation or recent extensive hybridization. If recent extensive hybridization has occurred among populations because of effective gene flow commonly observed in wind-pollinated conifers, then the genetic data would fit the *f* = 0 better than the full model in HICKORY, reaching migration-drift equilibrium and displaying limited LD. Moreover, the recalcitrant seed storage behavior [29] and low regeneration rate in natural stands [30] of *K. davidiana* var. *formosana* may have substantially restricted seed dispersal. Therefore, recent extensive hybridization due to high levels of gene flow is unlikely, suggesting that the retention of historically more-widespread ancestral polymorphisms may have been the reason for the current admixture of individual genotypes among populations, especially between the disjunctly distributed northern and southern populations. However, rare long-distance dispersals especially via pollen flow cannot completely be discounted [72].

High levels of genetic differentiation in all hierarchical population groups based on the outlier dataset are potentially results from recent bottleneck inflicted by human disturbances. The bottleneck may have caused inbreeding and genetic isolation in *K. davidiana* var. *formosana* populations as suggested by the significant associations between alleles within populations according to significant *I*_A_ and *r*_d_ values.

### Divergent Selection and Local Adaptation

BAPS is known to be more efficient at identifying hidden population structures [50], and BAPS at *K* = 4 revealed a genetic subdivision within the DW41 population based on the total dataset in this study. However, with the outlier dataset, no genetic substructuring within the DW41 population was found. The underlying cause of the observed subdivision within the DW41 population based on the total dataset remains unclear; however, the finding of no subdivision based on the outlier dataset suggests that previous colonization of different ancestral lineages and later uniform, directional selection increased the fixation rate of a set of beneficial alleles which differed from those of other populations. The inference of directional selection can also be applied to the DW30 population, as analyzed using the outlier dataset by both STRUCTURE and BAPS at *K* = 4. The genetic distinction between the disjunctly distributed northern and southern populations and between the two southern populations revealed by Bayesian clustering in both STRUCTURE and BAPS at *K* = 4, based on the outlier dataset, suggest that divergent selection regimes might have been effectively acting on environmentally disparate populations for a long period of time [33], [88], [89], even initiated before the recent bottlenecks inflicted by human disturbances.

One locus with a log_10_(BF) of slightly larger than 0.5 in one population pair comparison can have a log_10_(BF) value of >1 in other population pair comparisons, and can be considered to be a “strong” or even “decisive” signature of selection according to Jeffreys’ scale of evidence [56]. Therefore, we considered a locus being an outlier with a log_10_(BF) >0.5 in BAYESCAN. It is possible that several factors can account for discrepant log_10_(BF) values obtained for the same specific outlier locus in different population pair comparisons, such as (i) different selection regimes acting between populations; (ii) different allele frequencies of genetic variation between populations; or (iii) different patterns of divergence hitchhiking between populations. Anonymous AFLP loci detected as outliers may represent genomic regions rather than specific variation under selection themselves [90]. Identification of these loci as outliers by both DFDIST and BAYESCAN provides additional support that they are located in or near genomic regions under selection [91].

It is not unexpected that no outliers were found in pairwise population comparisons of the three northern populations using both DFDIST and BAYESCAN, because of very low levels of genetic differentiation, their geographic proximity, and environmental homogeneity. However, divergent selection can be further inferred between the two southern populations and the three northern populations, because DFDIST and BAYESCAN identified a common outlier (locus 4) specific to all comparisons between the southern DW30 and the three northern populations, and one common outlier (locus 6) specific to all comparisons between the southern DW41 and the three northern populations.

The AFLP loci are not likely the loci that are under selection. More likely, they are linked to candidate genes. Three AFLP loci (loci 1, 4, and 5) can be considered strong candidates of selection, because they were identified by both the DFDIST and BAYESCAN tests under different assumptions, they are also potentially associated to ecological variables in the SAM and GEE analyses. Outliers that were not associated with environmental variables may simply be the results of divergence hitchhiking, because they are closely linked to loci directly selected by natural selection [92]–[94]. Divergence hitchhiking patterns that vary across populations can be inferred by the finding of differential patterns of LD between ecologically and non-ecologically associated outliers [95], [96]. This may have been promoted by a certain degree of non-random mating, suggested by the HICKORY and multilocus LD test results, consistent with a finding of a high cone abortion rate possibly due to inbreeding in *K. davidiana* var. *formosana* [97]. Moreover, differential patterns of LD between ecologically and non-ecologically associated outliers among populations are indicative of the potential evolution of locally co-adapted gene complexes, which may be facilitated by divergent selection and result in adaptive divergence [1], [33], [95], [96]. However, LD between outliers may have been caused by increased genetic drift due to bottlenecks [98], [99].

Local adaptation could be inferred because of identification of three ecologically associated outliers among environmentally disparate *K. davidiana* var. *formosana* populations, and because common ecologically associated outliers were identified when the two southern populations were independently compared to the northern ST population (loci 1 and 5). It is worth noting that ecologically associated AFLP loci 1 and 5 potentially exhibiting strong selection acted to segregate local adaptation at these outliers even while populations were more widespread, suggesting strong selection acting on the same genetic variation in closely related populations. This result of repeated selection of the same ecologically associated outliers suggests that common selection regimes in distinct environments have played important roles in local adaptation.

### Conclusions

The AFLP data found a relatively high level of genetic diversity on average within *K. davidiana* var. *formosana*, despite its population size decline due to deforestation. However, *K. davidiana* var. *formosana* seems to be genetically endangered because of population size reductions that may have generated significant within population LD and inbreeding. The high level of genetic differentiation based on outlier loci suggests a divergence and selection process, resulting in local adaptation of *K. davidiana* var. *formosana* populations. The two southern populations should perhaps be used as seed sources for future population reestablishment. Information obtained in this study is essential to guide policy of management and conservation action for this critically endangered species. More studies can be performed to test for environmentally associated adaptive divergence using high-throughput sequencing technologies at the level of whole genome [100]–[102]. This type of study would assist in understanding what genes may actually involve in divergent selection and local adaptation beyond the anonymous AFLP markers.

## Supporting Information

Table S1 **Primer combinations and sequences of the three bases additional to the** ***Eco*** **RI (5′GACTGCGTACCAATTC3′) or** ***Mse*** **I (5′GATGAGTCCTGAGTAA3′) adaptor used for AFLP analysis.**
(DOC)

Table S2 **Assessment of differences in environmental variables among populations of** ***Keteleeria davidiana*** **var.** ***formosana*** **using a multivariate analysis of variance.**
(DOC)

Table S3 **Detection of outliers using DFDIST, BAYESCAN, both DFDIST and BAYESCAN in a variety of population pairwise comparisons, and SAM and GEE in total population.**
(DOC)

## References

[1] Kawecki TJ, Ebert D (2004) Conceptual issues in local adaptation. Ecol Lett 7: 1225–1241.

[2] Hedrick PW (2006) Genetic polymorphism in heterogeneous environments: The age of genomics. Annu Rev Ecol Evol System 37: 67–93.

[3] Black WC, Baer CF, Antolin MF, DuTeau NM (2001) Population genomics: genome-wide sampling of insect populations. Annu Rev Entomol 46: 441–469. 11112176 10.1146/annurev.ento.46.1.441

[4] Schlötterer C (2003) Hitchhiking mapping-functional genomics from the population genetics perspective. Trends Genet 19: 32–38. 12493246 10.1016/s0168-9525(02)00012-4

[5] Bridle JR, Vines TH (2006) Limits to evolution at range margins: when and why does adaptation fail? Trends Ecol Evol 22: 140–147. 17113679 10.1016/j.tree.2006.11.002

[6] Leimu R, Fischer M (2008) A meta-analysis of local adaptation in plants. PLoS ONE 3: e4010. 19104660 10.1371/journal.pone.0004010 PMC2602971

[7] Herrera CM, Bazaga P (2009) Quantifying the genetic component of phenotypic variation in unpedigreed wild plants: tailoring genomic scan for within-population use. Mol Ecol 18: 2602–2614. 19457184 10.1111/j.1365-294X.2009.04229.x

[8] Meyer CL, Vitalis R, Saumitou-Laprade P, Castric V (2009) Genomic pattern of adaptive divergence in *Arabidopsis halleri*, a model species for tolerance to heavy metal. Mol Ecol 18: 2050–2062. 19434814 10.1111/j.1365-294x.2009.04159.x

[9] Luikart G, England PR, Tallmon D, Jordan S, Taberlet P (2003) The power and promise of population genomics: from genotyping to genome typing. Nat Rev Genet 4: 981–994. 14631358 10.1038/nrg1226

[10] Storz JF (2005) Using genome scans of DNA polymorphism to infer adaptive population divergence. Mol Ecol 14: 671–688. 15723660 10.1111/j.1365-294X.2005.02437.x

[11] Bensch S, Akesson M (2005) Ten years of AFLP in ecology and evolution: why so few animals? Mol Ecol 14: 2899–2914. 16101761 10.1111/j.1365-294X.2005.02655.x

[12] Beaumont MA, Balding DJ (2004) Identifying adaptive genetic divergence among populations from genome scans. Mol Ecol 13: 969–980. 15012769 10.1111/j.1365-294x.2004.02125.x

[13] Beaumont MA, Nichols RA (1996) Evaluating loci for use in the genetic analysis of population structure. Proc Roy Soc B-Biol Sci 263: 1619–1626.

[14] Foll M, Gaggiotti O (2008) A genome scan method to identify selected loci appropriate for both dominant and codominant markers: a Bayesian perspective. Genetics 180: 977–993. 18780740 10.1534/genetics.108.092221 PMC2567396

[15] Pérez-Figueroa A, Garcia-Pereira MJ, Saura M, Rolán-Alvarez E, Caballero A (2010) Comparing three different methods to detect selective loci using dominant markers. J Evol Bio 23: 2267–2276. 20796133 10.1111/j.1420-9101.2010.02093.x

[16] Jump AS, Hunt JM, Martinez-Izquierdo JA, Peñuelas J (2006) Natural selection and climate change: temperature-linked spatial and temporal trends in gene frequency in *Fagus sylvatica* . Mol Ecol 15: 3469–3480. 16968284 10.1111/j.1365-294X.2006.03027.x

[17] Joost S, Bonin A, Bruford MW, Després L, Conord C, et al (2007) A spatial analysis method (SAM) to detect candidate loci for selection: towards a landscape genomics approach to adaptation. Mol Ecol 16: 3955–3969. 17850556 10.1111/j.1365-294X.2007.03442.x

[18] Poncet BN, Herrmann D, Gugerli F, Taberlet P, Holderegger R, et al (2010) Tracking genes of ecological relevance using a genome scan in two independent regional population samples of *Arabis alpine* . Mol Ecol 19: 2896–2907. 20609082 10.1111/j.1365-294X.2010.04696.x

[19] Manel S, Gugerli F, Thuiller W, Alvarez N, Legendre P, et al (2012) Broad-scale adaptive genetic variation in alpine plants is driven by temperature and precipitation. Mol Ecol 21: 3729–3738. 22680783 10.1111/j.1365-294X.2012.05656.x PMC4003392

[20] Despres L, Loriot S, Gaudeul M (2002) Geographic pattern of genetic variation in the European globeflower *Trollius europaeus* L. (Ranunculaceae) inferred from amplified fragment length polymorphisms. Mol Ecol 11: 2337–2347. 12406244 10.1046/j.1365-294x.2002.01618.x

[21] Bonin A, Miaud C, Taberlet P, Pompanon F (2006) Explorative genome scan to detect candidate loci for adaptation along a gradient of altitude in the common frog (*Rana temporaria*). Mol Biol Evol 23: 773–783. 16396915 10.1093/molbev/msj087

[22] Nunes VL, Beaumont MA, Butlin RK, Paulo OS (2011) Multiple approaches to detect outliers in a genome scan for selection in ocellated lizards (*Lacerta lepida*) along an environmental gradient. Mol Ecol 20: 193–205. 21091562 10.1111/j.1365-294X.2010.04936.x

[23] Bothwell H, Bisbing S, Therkildsen NO, Crawford L, Alvarez N, et al (2013) Identifying genetic signatures of selection in a non-model species, alpine gentian (*Gentiana nivalis* L.), using a landscape genetic approach. Conser Genet 14: 467–481.

[24] Li HL, Hsuan K (1994) Pinaceae. In: Editorial Committee of the Flora of Taiwan, editor. Flora of Taiwan (second edition). Taipei: Taiwan. 568–569.

[25] Farjon A (1989) A second revision of the genus *Keteleeria Carrière* (Taxonomic notes on Pinaceae II). Notes Roy Bot Gard Edinburgh 46: 81–99.

[26] Tsukada M (1967) Vegetation in subtropical Formosa during the Pleistocene glaciations and the Holocene. Palaeogeo Palaeoclima Palaeoecol 3: 49–64.

[27] Liew PM, Chung NJ (2001) Vertical migration of forests during the last glacial period in subtropical Taiwan. West Pac Earth Sci 1: 405–414.

[28] Su H (1984) Studies on the climate and vegetational types of the natural forest in Taiwan (II): altitudinal variation in zones in relation to temperature gradient, Q J Chin For. 17: 57–73.

[29] Yang J-C, Lin TP, Kuo S-R (2006) Seed storage behavior of Taiwan cow-tail fir (*Ketelleria davidiana* (Franchet) Beissner var. *formosana* Hayata). Taiwan J For Sci 21: 179–189.

[30] Wang YN (1987) Reproductive cycle and some anatomical studies on *Keteleeria fomosana* Hay. PhD thesis. Department of Forestry, National Taiwan University.

[31] Kanehira R (1936) Formosan trees indigenous to the island (revised). Taihoku, Formosa [Taiwan]: Department of Forestry, Government Research Institute. 51.

[32] Chou FS, Yang CK, Lin WL, Chen TY, Yang YP, et al (2009) Syntaxonomic and gradient analysis of *Keteleeria davidiana* var. *formosana* forests in Taiwan. Taiwan J For Sci 24: 257–269.

[33] Rundle HD, Nosil P (2005) Ecological speciation. Ecol Lett 8: 336–352.

[34] Nosil P, Harmon LJ, Seehausen O (2009) Ecological explanations for (incomplete) speciation. Trends Ecol Evol 24: 145–156. 19185951 10.1016/j.tree.2008.10.011

[35] Via S (2009) Natural selection in action during speciation. Proc Natl Acad Sci USA 106: 9936–9946. 10.1073/pnas.0901397106 PMC2702801 19528641

[36] Holsinger KE, Lewis PO (2003) Hickory: a package for analysis of population genetic data v1.0 distributed by the authors, Department of Ecology and Evolutionary Biology, University of Connecticut, Storrs, USA. Available: http://darwin.eeb.uconn.edu/hickory/software.html.

[37] Halekoh U, Højsgaard S, Yan J (2006) The R package geepack for generalized estimating equations. J Stat Soft 15: 1–11.

[38] Vos P, Hogers R, Bleeker M, Reijans M, van de Lee T, et al (1995) AFLP: a new technique for DNA fingerprinting. Nucl Acids Res 23: 4407–4414. 7501463 10.1093/nar/23.21.4407 PMC307397

[39] Bonin A, Ehrich D, Manel S (2007) Statistical analysis of amplified fragment length polymorphism data: a toolbox for molecular ecologists and evolutionists. Mol Ecol 16: 3737–3758. 17850542 10.1111/j.1365-294X.2007.03435.x

[40] Carrera-Hernández JJ, Gaskin SJ (2007) Spatio temporal analysis of daily precipitation and temperature in the basin of Mexico. J Hydrol 336: 231–249.

[41] SAS Institute (2007) JMP version 7. SAS Institute Inc., Cary (NC): USA.

[42] Nei M (1973) Analysis of gene diversity in subdivided populations. Proc Natl Acad Sci USA 70: 3321–3323. 4519626 10.1073/pnas.70.12.3321 PMC427228

[43] Vekemans X, Beauwens T, Lemaire M, Roldán-Ruiz I (2002) Data from amplified fragment length polymorphism (AFLP) markers show indication of size homoplasy and of a relationship between degree of homoplasy and fragment size. Mol Ecol 11: 139–151. 11903911 10.1046/j.0962-1083.2001.01415.x

[44] Excoffier L, Lischer HEL (2010) Arlequin suite ver 3.5: A new series of programs to perform population genetics analyses under Linux and Windows. Mol Ecol Res 10: 564–567. 10.1111/j.1755-0998.2010.02847.x 21565059

[45] Pritchard JK, Stephens M, Donnelly P (2000) Inference of population structure using multilocus genotype data. Genetics 155: 945–959. 10835412 10.1093/genetics/155.2.945 PMC1461096

[46] Falush D, Stephens M, Pritchard JK (2007) Inference of population structure using multilocus genotype data: dominant markers and null alleles. Mol Ecol Notes 7: 574–578. 18784791 10.1111/j.1471-8286.2007.01758.x PMC1974779

[47] Corander J (2004) BAPS 2: enhanced possibilities for the analysis of genetic population structure. Bioinformatics 20: 2363–2369. 15073024 10.1093/bioinformatics/bth250

[48] Corander J, Marttinen P, Sirén J, Tang J (2008) Enhanced Bayesian modelling in BAPS software for learning genetic structures of populations. BMC Bioinformatics 9: 539. 19087322 10.1186/1471-2105-9-539 PMC2629778

[49] Corander J Waldmann P, Sillanpaa MJ (2003) Bayesian analysis of genetic differentiation between populations. Genetics 163: 367–374. 12586722 10.1093/genetics/163.1.367 PMC1462429

[50] Corander J, Marttinen P (2006) Bayesian identification of admixture events using multilocus molecular markers. Mol Ecol 15: 2833–2843. 16911204 10.1111/j.1365-294X.2006.02994.x

[51] Evanno G, Regnaut S, Goudet J (2005) Detecting the number of clusters of individuals using the software STRUCTURE: a simulation study. Mol Ecol 14: 2611–2620. 15969739 10.1111/j.1365-294X.2005.02553.x

[52] Holsinger KE, Lewis PO, Dey DK (2002) A Bayesian approach to inferring population structure from dominant markers. Mol Ecol 11: 1157–1164. 12074723 10.1046/j.1365-294x.2002.01512.x

[53] Holsinger KE, Wallace LE (2004) Bayesian approaches for the analysis of population structure: an example from *Platanthera leucophaea* (Orchidaceae). Mol Ecol 13: 887–894. 15012763 10.1111/j.1365-294x.2004.02052.x

[54] Zhivotovsky (1999) Estimating population structure in diploids with multilocus dominant DNA markers. Mol Ecol 8: 907–913. 10434412 10.1046/j.1365-294x.1999.00620.x

[55] Benjamini Y, Hochberg Y (1995) Controlling the false discovery rate: a practical and powerful approach to multiple testing. J Roy Statist Soc B (Methodological) 57: 289–300.

[56] Jeffreys H (1961) Theory of probability (third edition). Oxford: Oxford University Press.

[57] Joost S, Kalbermatten M, Bonin A (2008) Spatial Analysis Method (SAM): a software tool combining molecular and environmental data to identify candidate loci for selection. Mol Ecol Res 8: 957–960. 10.1111/j.1755-0998.2008.02162.x 21585940

[58] Agapow PM, Burt A (2001) Indices of multilocus linkage disequilibrium. Mol Ecol Notes 1: 101–102.

[59] Brown AHD, Feldman MW, Nevo E (1980) Multilocus structure of natural populations of *Hordeum spontaneum* . Genetics 96: 523–536. 17249067 10.1093/genetics/96.2.523 PMC1214315

[60] Maynard SJ, Smith NH, O’Rourke M, Spratt BG (1993) How clonal are bacteria? Proc Natl Acad Sci USA 90: 4384–4388. 8506277 10.1073/pnas.90.10.4384 PMC46515

[61] Haubold B, Travisano M, Rainey PB, Hudson RR (1998) Detecting linkage disequilibrium in bacterial populations. Genetics 150: 1341–1348. 9832514 10.1093/genetics/150.4.1341 PMC1460433

[62] Hamrick JL, Godt MJW (1996) Conservation genetics of endemic plant species. In: Avise JC, Hamrick JL, editors. Conservation genetics. New York: Chapman and Hall. 281–304.

[63] Lenormand T (2002) Gene flow and the limits to natural selection. Trends Ecol Evol 17: 183–189.

[64] Goldberg EE, Lande R (2007) Species’ borders and dispersal barriers. Am Natur 170: 297–304. 17874380 10.1086/518946

[65] Chung JD, Lin TP, Tan YC, Lin MY, Hwang SY (2004) Genetic diversity and biogeography of *Cunninghamia konishii* (Cupressaceae), an island species in Taiwan: a comparison with *Cunninghamia lanceolata*, a mainland species in China. Mol Phylogent Evol 33: 791–801. 10.1016/j.ympev.2004.08.011 15522804

[66] Terrab A, Hampe A, Lepais O, Talavera S, Vela E, et al (2008) Phylogeography of North African atlas cedar (*Cedrus atlantica*, Pinaceae): combined molecular and fossil data reveal a complex Quaternary history. Am J Bot 95: 1262–1269. 21632331 10.3732/ajb.0800010

[67] Tang S, Dai W, Li M, Zhang Y, Geng Y, et al (2008) Genetic diversity of relictual and endangered plant *Abies ziyuanensis* (Pinaceae) revealed by AFLP and SSR markers. Genetica 133: 21–30. 17661154 10.1007/s10709-007-9178-x

[68] Parchman TL, Benkman CW, Jenkins B, Buerkle CA (2011) Low levels of population genetic structure in *Pinus contorta* (Pinaceae) across a geographic mosaic of co-evolution. Am J Bot 98: 669–679. 21613166 10.3732/ajb.1000378

[69] Nybom H (2004) Comparison of different nuclear DNA markers for estimating intraspecific genetic diversity in plants. Mol Ecol 13: 1143–1155. 15078452 10.1111/j.1365-294X.2004.02141.x

[70] Bradshaw AD (1984) Ecological significance of genetic variation between populations. In: Dirzo R, Sarukhán J, editors. Perspectives on plant population ecology. Sunderland (MA): Sinauer Associates. 213–228.

[71] Reed DH, Frankham R (2003) Correlation between fitness and genetic diversity. Conserv Biol 17: 230–237.

[72] Petit RJ, Hampe A (2006) Some evolutionary consequences of being a tree. Annu Rev Ecol Evol Syst 37: 187–214.

[73] Barrett RDH, Schluter D (2008) Adaptation from standing genetic variation. Trends Ecol Evol 23: 38–44. 18006185 10.1016/j.tree.2007.09.008

[74] Brown GR, Gill GP, Kuntz RJ, Langley CH, Neale DB (2004) Nucleotide diversity and linkage disequilibrium in loblolly pine. Proc Natl Acad Sci USA 101: 15255–15260. 15477602 10.1073/pnas.0404231101 PMC524047

[75] Heuertz M, De Paoli E, Kallman T, Larsson H, Jurman I, et al (2006) Multilocus patterns of nucleotide diversity, linkage disequilibrium and demographic history of Norway spruce [*Picea abies* (L.) Karst]. Genetics 174: 2095–2105. 17057229 10.1534/genetics.106.065102 PMC1698656

[76] Eckert AJ, Bower AD, González-Martínez SC, Wegrzyn JL, Coop G, et al (2010) Back to nature: Ecological genomics of loblolly pine (*Pinus taeda*, Pinaceae). Mol Ecol 19: 3789–3805. 20723060 10.1111/j.1365-294X.2010.04698.x

[77] Pyhäjärvi T, Kujala ST, Savolainen O (2011) Revisiting protein heterozygosity in plants-nucleotide diversity in allozyme coding genes of conifer *Pinus sylvestris* . Tree Genet Gen 7: 385–397.

[78] Hedrick PW (2000) Genetics of populations, Sudbury: Jones and Bartlett Publishers.

[79] Hill WG, Robertson A (1968) Linkage disequilibrium in finite populations. Theor Appl Genet 38: 226–231. 24442307 10.1007/BF01245622

[80] Ohta T, Kimura M (1969) Linkage disequilibrium at steady state determined by random genetic drift and recurrent mutation. Genetics 63: 229–238. 5365295 10.1093/genetics/63.1.229 PMC1212334

[81] Wright AF, Carothers AD, Pirastu M (1999) Population choice in mapping genes for complex diseases. Nat Genet 23: 397–404. 10581024 10.1038/70501

[82] Nei M, Maruyama T, Chakraborty R (1975) The bottleneck effect and genetic variability in populations. Evolution 29: 1–10. 28563291 10.1111/j.1558-5646.1975.tb00807.x

[83] Maruyama T, Fuerst PA (1985) Population bottlenecks and nonequilibrium models in population genetics. II. Number of alleles in a small population that was formed by a recent bottleneck. Genetics 11: 675–689. 10.1093/genetics/111.3.675 PMC1202664 4054612

[84] Wright SI, Gaut BS (2005) Molecular population genetics and the search for adaptive evolution in plants. Mol Biol Evol 22: 506–519. 15525701 10.1093/molbev/msi035

[85] Hamrick JL, Godt MJW, Sherman-Broyles SL (1992) Factors inﬂuencing levels of genetic diversity in woody plant species. New For 6: 95–124.

[86] Petit RJ, Deguilloux MF, Chat J, Grivet D, Garnier-Géré P, et al (2005) Standardizing for microsatellite length in comparisons of genetic diversity. Mol Ecol 14: 885–890. 15723680 10.1111/j.1365-294X.2005.02446.x

[87] Latch EK, Dharmarajan G, Glaubitz JC, Rhodes OE Jr (2006) Relative performance of Bayesian clustering software for inferring population substructure and individual assignment at low levels of population differentiation. Conser Genet 7: 295–302.

[88] Räsänen K, Hendry AP (2008) Disentangling interactions between adaptive divergence and gene flow when ecology drives diversification. Ecol Lett 11: 624–636. 18384363 10.1111/j.1461-0248.2008.01176.x

[89] Elias M, Faria R, Gompert Z, Hendry A (2012) Factors influencing progress toward ecological speciation. Inter J Ecol doi: 10.1155/2012/280169.

[90] Jensen JD, Wong A, Aquadro CF (2007) Approaches for identifying targets of positive selection. Trends Genet 23: 568–577. 17959267 10.1016/j.tig.2007.08.009

[91] Vasemägi A, Nilsson J, Primmer CR (2005) Expressed sequence tag-linked microsatellites as a source of gene-associated polymorphisms for detecting signatures of divergent selection in Atlantic salmon (*Salmo salar* L.). Mol Ecol 22: 1067–1076. 10.1093/molbev/msi093 15689532

[92] Wolf JBW, Lindell J, Backström N (2010) Speciation genetics: current status and evolving approaches. Philo Trans Roy Soc B-Biol Sci 365: 1717–1733. 10.1098/rstb.2010.0023 PMC2871893 20439277

[93] Feder JL, Egan SP, Nosil P (2012) The genomics of speciation-with-gene-flow. Trends Genet 28: 342–350. 22520730 10.1016/j.tig.2012.03.009

[94] Via S (2012) Divergence hitchhiking and the spread of genomic isolation during ecological speciation-with-gene-flow. Philo Trans Roy Soc B-Biol Sci 367: 451–460. 10.1098/rstb.2011.0260 PMC3233719 22201174

[95] Nosil P, Egan SP, Funk DJ (2007) Heterogeneous genomic differentiation between walking-stick ecotypes: “Isolation by adaptation” and multiple roles for divergence. Evolution 62: 316–336. 17999721 10.1111/j.1558-5646.2007.00299.x

[96] Hohenlohe PA, Bassham S, Curry M, Cresko WA (2012) Extensive linkage disequilibrium and parallel adaptive divergence across threespine stickleback genomes. Philos Trans R Soc B-Biol Sci 367: 395–408. 10.1098/rstb.2011.0245 PMC3233713 22201169

[97] Hung SC, Lin HC, Li MJ, Chung JD (2004) The restoration of *Keteleeria davidiana* (Franchet) Beissner var. *formosana* . Taiwan For J 13: 50–57.

[98] Przeworski M (2002) The signature of positive selection at randomly chosen loci. Genetics 160: 1179–1189. 11901132 10.1093/genetics/160.3.1179 PMC1462030

[99] Wall JD, Andolfatto P, Przeworski M (2002) Testing models of selection and demography in *Drosophila simulans* . Genetics 162: 203–216. 12242234 10.1093/genetics/162.1.203 PMC1462246

[100] Manel S, Joost S, Epperson BK, Holderegger R, Storfer A, et al (2010) Perspectives on the use of landscape genetics to detect genetic variation in the field. Mol Ecol 19: 3760–3772. 20723056 10.1111/j.1365-294X.2010.04717.x

[101] Barrett RDH, Hoekstra H (2011) Molecular spandrels: tests of adaptation at the genetic level. Nature Rev Genet 12: 767–780. 22005986 10.1038/nrg3015

[102] Hansen MM, Olivieri I, Waller DM, Nielsen EE (2012) The GeM Working group (2012) Monitoring adaptive genetic responses to environmental change. Mol Ecol 21: 1311–1329. 22269082 10.1111/j.1365-294X.2011.05463.x

