# Supplementary material for: Divergent Selection and Local Adaptation in Disjunct Populations of an Endangered Conifer, Keteleeria davidiana var. formosana (Pinaceae)
Source: PLoS One. 2013 Jul 22;8(7):e70162. doi: 10.1371/journal.pone.0070162 (PMC3718774; doi:10.1371/journal.pone.0070162)
Supplement: Table S1 — Primer combinations and sequences of the three bases additional to the Eco RI (5′GACTGCGTACCAATTC3′) or Mse I (5′GATGAGTCCTGAGTAA3′) adaptor used for AFLP analysis. (DOC) [file pone.0070162.s001.doc]

**Table S1** **Primer combinations and sequences of the three bases additional to the *Eco*RI (5’GA**

**CTGCGTACCAATTC3’) or *Mse*I (5’GATGAGTCCTGAGTAA3’) adaptor used for the AFLP analysis**.

| Primer | *Eco*RI | *Mse*I | Number of markers | Error rate |
| --- | --- | --- | --- | --- |
| 1 | AGT | ACT | 34 | 0.032 |
| 2 | CTA | ACT | 39 | 0.038 |
| 3 | AGT | TAG | 36 | 0.043 |
| 4 | CTA | TAG | 50 | 0.040 |
| 5 | AGT | TGT | 21 | 0.037 |
| 6 | CTA | TGT | 28 | 0.040 |
| 7 | AGT | CTA | 61 | 0.039 |
| 8 | CTA | CTA | 46 | 0.040 |
| 9 | AGT | GAT | 31 | 0.035 |
| 10 | CTA | GAT | 39 | 0.043 |
| 11 | AGT | GTA | 51 | 0.039 |
| 12 | CTA | GTA | 29 | 0.035 |
| Average |  |  | 38.75 | 0.038 |
