# Supplementary material for: Divergent Selection and Local Adaptation in Disjunct Populations of an Endangered Conifer, Keteleeria davidiana var. formosana (Pinaceae)
Source: PLoS One. 2013 Jul 22;8(7):e70162. doi: 10.1371/journal.pone.0070162 (PMC3718774; doi:10.1371/journal.pone.0070162)
Supplement: Table S2 — Assessment of differences in environmental variables among populations of Keteleeria davidiana var. formosana using a multivariate analysis of variance. (DOC) [file pone.0070162.s002.doc]

**Table S2 Assessment of differences in environmental variables among**

| Environmental variable | *F* | *P* |
| --- | --- | --- |
| Tmean (°C) | F_4, 55_ = 1.565 | 0.196 |
| Tmax (°C) | F_4, 55_ = 0.521 | 0.72 |
| Tmin (°C) | F_4, 55_ = 1.057 | 0.387 |
| WSmean (m/s) | F_4, 55_ = 0.64 | 0.636 |
| PRE (mm/month) | F_4, 55_ = 2.099 | 0.0934 |
| RH (%) | F_4, 55_ = 28.55 | 7.41e-13 |
| CLO (1~10) | F_4,55_ = 7.545 | 6.41e-05 |
| SunH (h) | F_4, 55_ = 4.594 | 0.0029 |
| D_30_ (no.) | F_4,55_ = 0.685 | 0.606 |
| D_10_ (no.) | F_4, 55_ = 2.159 | 0.0858 |
| RainD (d/month) | F_4, 55_ = 21.66 | 9e-11 |
| Total | F_44, 192_ = 3.5199 | 1.03e-09 |
| Tmean, mean temperature; Tmax, maximum temperature; Tmin, minimum temperature; WSmean, mean wind speed; PRE, precipitation; RH, relative humidity; CLO, cloud cover; SunH, time of sunshine; D_30_, days of maximum temperature > 30 °C; D_10_, days of minimum temperature < 10°C and RainD, wet days (number of days with >0.1 mm of rain per month). | | |

**populations of *Keteleeria davidiana* var. *formosana* using a multivariate analysis of variance.**
