# Supplementary material for: Divergent Selection and Local Adaptation in Disjunct Populations of an Endangered Conifer, Keteleeria davidiana var. formosana (Pinaceae)
Source: PLoS One. 2013 Jul 22;8(7):e70162. doi: 10.1371/journal.pone.0070162 (PMC3718774; doi:10.1371/journal.pone.0070162)
Supplement: Table S3 — Detection of outliers using DFDIST, BAYESCAN, both DFDIST and BAYESCAN in a variety of population pairwise comparisons, and SAM and GEE in total population. (DOC) [file pone.0070162.s003.doc]

**Table S3** **Detection of outliers using DFDIST, BAYESCAN, both DFDIST and BAYESCAN in a variety of population pairwise comparisons, and SAM and GEE in total population.**

| Comparison | Outliers | | | | | |
| --- | --- | --- | --- | --- | --- | --- |
|  | DFDIST | BAYESCAN | Both DFDIST and BAYESCAN | SAM | GEE | |
|  |  |  |  |  | PC1 | PC2 |
| Southern DW30 vs. all three northern populations | 3, 4, 5, 8, 10, 12, 15 | 4 | 4 | 1, 3, 4, 5, 8,10, 15, 101, 152, 409, 426 | 1, 4, 5, 13, 15, 32, 49, 56, 57, 65, 83, 96, 97, 98, 99, 101, 103, 104,107, 108, 109, 133, 134, 139, 152, 181, 183, 204, 210, 213, 218, 226, 234,238, 245, 254, 289, 335, 347, 356, 395, 400, 404, 405, 406, 409, 414, 416, 421, 425, 426, 430, 443, 459 | 33, 34, 39, 79, 81, 84, 87, 89, 115, 118, 124, 125, 136, 157, 192, 224, 242, 256, 265, 281, 282, 296, 303, 316, 328, 336, 337, 343, 353, 372, 376, 435, 437, 464 |
|  |  |  |  |  |  |  |
| Southern DW41 vs. all three northern populations | 6, 7, 9, 11 | 6 | 6 |  |  |  |
|  |  |  |  |  |  |  |
| Southern DW30 and DW41 vs. northern JGL | 2, 3, 4 | 2, 3 | 2, 3 |  |  |  |
|  |  |  |  |  |  |  |
| Southern DW30 and DW41 vs. northern GPL | 10, 14, 15, 16 | None | None |  |  |  |
|  |  |  |  |  |  |  |
| Southern DW30 and DW41 vs. northern ST | 1, 3, 4, 5, 8, 13 | 1, 3, 5 | 1, 3, 5 |  |  |  |
|  |  |  |  |  |  |  |
| Southern DW30 vs. southern DW41 | 6, 9, 17, 18, 19, 20, 21, 22, 23, 24, 25, 63, 115, 118, 136, 170, 180, 281, 358, 363, 364, 366, 369, 377, 379, 383, 385, 394, 399, 435, 437, 438, 460 | 17, 18, 19, 20, 21, 22, 23, 24, 25 | 17, 18, 19, 20, 21, 22, 23, 24, 25 |  |  |  |
